# Factors influencing health-related quality of life in children with asthma: insights from Addis Ababa public hospitals

**DOI:** 10.3389/fpubh.2024.1478707

**Published:** 2025-01-07

**Authors:** Tsehaynew Kasse, Selemaye Zenebe, Yalemzer Agegnehu, Arega Abebe Lonsako

**Affiliations:** College of Medicine and Health Sciences, Arba Minch University, Arba Minch, Ethiopia

**Keywords:** quality of life, asthma, children, Addis Ababa, Ethiopia

## Abstract

**Background:**

Bronchial asthma is a global health problem in particular a respiratory condition characterized by broncho spasms that negatively affect the quality of life (QOL) of children. However, there is a paucity of data regarding the health-related quality of life of asthma in children in Ethiopia, and the study area.

**Objective:**

The objective of this study was to assess the health-related quality of life among asthmatic children aged 7–17 in selected hospitals in Addis Ababa, Ethiopia.

**Methods:**

An institutional-based analytical cross-sectional study involving 136 asthmatic children aged 7–17 years was conducted in the selected hospital in Addis Ababa, from February to April 2024. Respondents were chosen using a systematic random sampling method. Structured, interviewer-administered, and pretested questionnaires, were used to collect data. The data were coded and entered into Epi-Data 3.1 before being exported to SPSS version 25 for analysis. Logistic regression was employed to identify factors influencing health-related quality of life Statistical significance was set at *p* < 0.05 with a 95% confidence interval.

**Results:**

The study found that 46% [95% CI: 37.6–54.4%] of the study participants had a poor quality of life. Factors associated with an increased likelihood of poor quality of life included caregivers’ lack of formal education (Adjusted Odds Ratio [AOR]: 1.39 [1.80–10.69]), a family history of asthma (AOR: 2.51 [1.46–4.299]), longer asthma duration (AOR: 3.47 [1.89–6.39]), uncontrolled asthma (AOR: 3.47 [1.89–6.39]), moderate persistent asthma (AOR: 2.4 [1.40–4.20]), and comorbidities (AOR: 2.4 [1.40–4.20]).

**Conclusion:**

The study highlights almost half of asthmatic children had a poor quality of life in Addis Ababa. Factors such as caregivers’ lack of formal education, a family history of asthma, longer duration and increased severity of asthma, uncontrolled asthma, and comorbidities were significantly associated with poor quality of life. Therefore, implementing targeted education programs, encouraging family history assessments, and strengthening comorbidity screening and management for children and their families in Addis Ababa are recommended.

## Introduction

Asthma, a chronic inflammatory disorder of the airways, poses a significant global health challenge, particularly among children due to its impact on respiratory function, which leads to variable airflow limitation and airway hyper-responsiveness (1–3). While asthma is both preventable and treatable, its prevalence and impact remain substantial worldwide, especially in pediatric populations where the burden is acutely felt (2, 4).

Over recent decades, there has been progress in reducing asthma morbidity and mortality. Between 1990 and 2019, morbidity rates declined from 601.20 to 477.92 per 100,000, while asthma-related mortality dropped from 8.60 to 5.96 per 100,000, indicating improved management, awareness, and treatment access (5).

Despite these gains, asthma still impacts millions, with an estimated 2.7 million children under 18 affected in the past year alone. This ongoing prevalence drives health inequities, particularly in resource-limited settings where children with asthma face frequent school absenteeism, increased medical visits, and recurrent hospitalizations (6, 7).

Although only 10% of pediatric asthma cases involve life-threatening complications, asthma accounts for a significant share of hospitalizations, contributing to 35% of overall hospital admissions and 77% of total hospitalization days (6, 7). Furthermore, around 25–66% of asthma cases in early childhood persist into adulthood, highlighting the long-term implications of this disease (8). In 2018, asthma affected an estimated 339 million people worldwide, ranking as the 14th leading cause of disability due to its high prevalence, in Africa, prevalence rates among children range widely, from 9% in Ethiopia to 20% in South Africa, underscoring the disease’s significant impact on children across the continent (9).

The burden of asthma extends beyond physical health, as it negatively impacts children’s lifestyles by limiting social interactions, cognitive development, and academic performance (7). Poor asthma management, often evidenced by frequent exacerbations and hospitalizations, correlates strongly with diminished quality of life, especially among children with severe airflow limitations or comorbid conditions like allergic rhinitis (10).

In developing nations, including Ethiopia, asthma often lacks prioritization within healthcare systems, leaving many patients without access to essential medications and comprehensive care (11). In 2015, asthma contributed to a mortality rate of 14.7 per 100,000 in Ethiopia, making it one of the country’s top 20 causes of death (12). Despite this significant health impact, there remains a scarcity of studies evaluating the health-related quality of life (HRQoL) among asthmatic children in Ethiopia. Quality of life is a multifaceted concept encompassing physical and mental health, personal relationships, educational and work environments, social status, financial security, freedom, safety, and physical surroundings (13).

This research aims to address this gap by assessing the health-related quality of life and its associated factors among asthmatic children at selected public hospitals in Addis Ababa, in 2024.

## Methods and materials

### Study area and period

The study was conducted in Addis Ababa, the capital city of Ethiopia, with an estimated population size of 5,703,628 in 2024. Addis Ababa is the 4th highest capital in the world. Located at the foot of Mount Entoto, at an altitude of 2,355 meters above sea level. According to the data obtained from Addis Ababa City Administration Health Bureau (14), there are 13 public hospitals in Addis Ababa, which were giving different services to the public. Three hospitals (Tikur Anbessa Specialized Hospital, Zewditu Memorial Hospital, and St. Paulo’s Hospital) were randomly selected to be the target public hospitals. The study was conducted from February to April 2024.

### Study design

A facility-based analytical cross-sectional study was employed.

### Population

#### Source population

All asthmatic children aged 7–17 years old who attend follow-up in outpatient and inpatient departments in the selected hospitals in Addis Ababa City.

#### Study population

All asthmatic children aged 7–17 years old who fulfilled inclusion criteria.

### Inclusion criteria and exclusion criteria

#### Inclusion criteria

All children aged between 7 and 17 who had been diagnosed with asthma were on constant follow-up in outpatient and inpatient departments for 3 months.

#### Exclusion criteria

Patients who had no caregiver and were unable to respond due to physical and proven mental problems were excluded from the study.

### Sample size determination

The sample size for this study was calculated using the single population proportion formula:


$$n={\left(Z\alpha /2\right)}^{2*}\;p^{*}\;\left(1-p\right)/d^{2}$$

Where *p* was set at 50% due to the absence of prior estimates for this age group in similar settings, *d* represented a 5% margin of error, and *Zα*/2 was 1.96 for a 95% confidence interval. Substituting these values, the initial sample size *n* was computed as 384. Given that the average monthly population of children in the selected hospitals was 170, a finite population correction was applied using the formula:


$$Ni=\text{No}/\left(1+\text{No}/N\right)$$

Where *No* is the initial sample size (384) and *N* is the total population (170). This calculation yielded a corrected sample size of approximately 136 participants. Thus, the final sample size determined for the study was 136.

### Sampling technique/procedure

**Figure 1:**
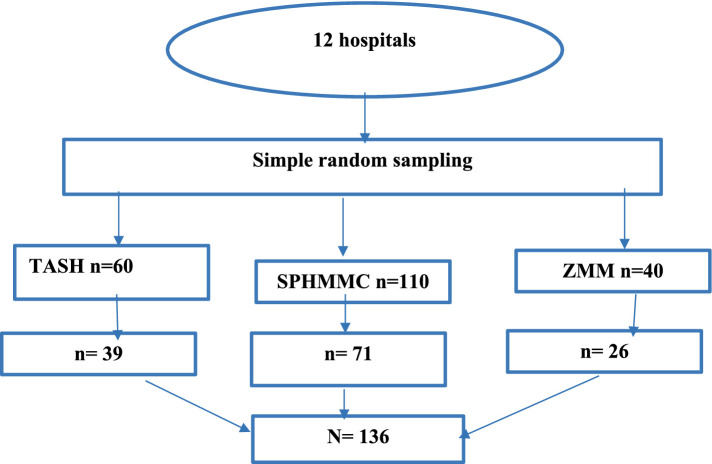
Schematic presentation of sampling procedures for the assessment of health-related quality of life and associated factors among asthmatic children at selected public hospitals, Addis Ababa, Ethiopia, 2024.

A simple random sampling technique was employed to select three hospitals. The sample size was proportionally allocated to each hospital based on the total number of asthmatic children visited in the last 3 months before data collection. The total number of asthmatic children (*N*) was determined (210). A proportional allocation factor (Nh) was calculated (n/N). This factor (0.65) was multiplied by the number of patients in each hospital (ni) to determine the sample size allocated to each hospital (nh). To select the required sample, in each hospital, systematic sampling techniques using their registration numbers or roster First, the average number of patients visiting the selected hospital in a month has been determined which is 210. Every k interval is calculated, i.e., *k* = 210/136 = 2, so that every third patient chart is included until the required sample size is achieved. The first patient was taken using the lottery method (Figure 1).

### Study variables

#### Dependent variables

Quality of life among asthmatic children.

#### Independent variable

Socio-demographics-related categorical variables and a continuous age variable: Age, sex, educational status of caregiver, residence, body mass index, and occupation of caregiver.

Disease and parent-related categorical variables: Comorbidities, severity of asthma, duration of asthma, drug usage status, and family history of asthma.

### Level of asthma control

#### Operational definition

*Asthma control* was evaluated using a 5-point Likert-type rating scale, with scores ranging from 5 (indicating poor asthma control) to 25 (reflecting complete asthma control). Higher scores were indicative of better asthma control. A score of <19 points indicated uncontrolled asthma, while a score of ≥19 indicated controlled asthma (15, 16).

The Pediatric Asthma Quality of Life Questionnaire (PAQLQ) assesses children’s quality of life through 23 items rated on a 7-point scale (1 = severe impairment, 7 = no impairment). The overall PAQLQ score is the mean of all item responses, with scores above the mean indicating good quality of life and scores at or below the mean indicating poor quality of life. Domain scores are calculated similarly. Impairment levels are categorized as follows: scores of 6 or higher indicate minimal or no impairment, scores between 3 and 6 indicate moderate impairment, and scores below 3 indicate severe impairment for both overall and domain-specific assessments (16–18).

Drug usage was evaluated using a five-point Likert scale across six items, with a total score generated by summing responses for these items, the mean total score was then used as a cutoff: participants with scores above the mean were categorized as having appropriate drug usage, while those with scores at or below the mean were categorized as having inappropriate usage (16, 17).

#### Tools for data collection

The data for this study were collected using a structured, pretested interviewer-administered questionnaire consisting of five sections. The first section gathered socio-demographic characteristics of asthmatic children, including age, sex, level of education, occupation, income, and residence. The second section included the PAQLQ, a 23-item tool designed to assess the quality of life across domains such as symptoms, activity limitations, emotional function, and environmental stimuli. The third section focused on other factors associated with the quality of life of asthmatic children.

The fourth section employed the Asthma Control Tool (ACT), a validated instrument for assessing asthma control. It utilized a 5-point Likert scale to evaluate daytime and nocturnal symptoms, with scores ranging from 5 (poor control) to 25 (complete control), indicating better control with higher scores. The ACT demonstrated internal consistency, with a Cronbach’s alpha of 0.8, and the final section assessed drug usage-related experience among asthmatic children (15, 16).

#### Data collection procedure

Data collection was carried out through face-to-face interviews conducted by a team of three trained nurses, supervised by two MSc nurses. The principal investigator was responsible for overseeing all daily activities related to the data collection process. To ensure consistency and reduce measurement bias, a comprehensive one-day training session was provided to both the data collectors and supervisors. This training focused on effective interview techniques, ethical considerations, and the rights of participants, including their ability to choose whether to participate or withdraw from the study at any time.

#### Data quality control

Appropriately designed and validated data collection tools were used, and data collectors and supervisors got 1 day of intensive training on data collection methods and procedures. A pretest was conducted 2 weeks before the main data collection at Yekatit 12 Hospital before the data collection period, and any ambiguity, confusion, and difficult words were revised, the appropriateness of the tool was based on the pretest experience. Supervisors and investigators closely oversee the data collection processes daily. Investigators were checked for inconsistencies, and possible corrections were made during the data collection period. Study participants were interviewed in private to reduce social desirability bias.

#### Data processing and analysis

First, the data were coded and entered into EpiData version 3.1 and then it was exported to the SPSS version 25 statistical package for further analysis. Descriptive statistics and binary logistic regression analysis were done to analyze data. A Hosmer-Lemeshow test was used to test for model fitness with a value (0.45), and a Multi co-linearity test was carried out to see the correlation between independent variables using variance inflation factor, with the value (1.05–2.01). Variables with a *p*-value <0.25 in bivariate analysis were entered into multivariable analysis to control the confounding effect of other variables. Descriptive analysis such as mean, standard deviation, and frequency was used and the results were presented as proportion through tables, text, and graphs, the output of logistic regression, as COR to show the strength of association between independent variables and dependent variables. Adjusted Odds ratios (AOR) with a corresponding 95% confidence interval were estimated. And statistical significance was declared at *p*-values <0.05.

## Results

### Characteristics of study participants

This study involved 136 asthmatic children aged 7–17 years old which gave a 100% response rate. Over half of the children (52.9%) were male, with the mean age being 9.46 (±2.29) years. Among the caregivers, 42.6% had no formal education, the majority (79.4%) resided in urban areas, and 45.6% of children were underweight (Table 1).

**Table 1:** Sociodemographic characteristics of asthmatic children at selected public hospitals in Addis Ababa, Ethiopia, 2024 (*N* = 136).

Variable	Category	Frequency	Percentage
Sex	Male	72	52.9
	Female	64	47.1
Age	07-Oct	84	61.8
	Oct-14	40	29.4
	14–17	12	8.8
Level of caregiver education	No formal education	58	42.6
	Primary	42	30.9
	Secondary	20	14.7
	Above secondary	16	11.8
Address	Urban	108	79.4
	Rural	28	20.6
Caregiver/ parent occupation	Housewife	82	60.3
	Government employee	22	16.2
	Daily labor	4	2.9
	Merchant	6	4.4
	Private employee	22	16.2
Body mass index	Underweight	62	46
	Normal	60	44
	Overweight	14	10

### Duration, severity, and drug usage among asthma study participants

The study revealed that the average duration of asthma (SD) among the participants was 25 (14) months. Among the children, more than half of them (60.3%) were found to have mild persistent asthma, followed by 39.7% with moderate persistent asthma. Additionally, it was found that 57.4% of the children were using appropriately their medication, while 42.6% were not.

### Comorbid conditions and triggers among asthma of study participants

The study revealed that the most common comorbidities among the participants were allergic rhinitis (38.2%) and allergic dermatitis (29.4%). The most common triggers for asthma exacerbation were a combination of upper respiratory tract infections (URTI), dust, and cold weather (44.1%), followed by (URTI) (29.4%). Additionally, a quarter of the participants reported a family history of asthma (Table 2).

**Table 2:** Comorbid conditions and triggers of asthma of asthmatic children at selected public hospitals in Addis Ababa, Ethiopia, 2024 (*N* = 136).

Variable	Category	Frequency	Percentage
Family history	Yes	34	25
	No	102	75
Comorbid conditions	Allergic rhinitis only	52	38.2
	Atopic dermatitis only	40	29.4
	Allergic rhinitis and Atopic dermatitis	42	30.9
	No comorbidity	2	1.5
Triggers	URTI	40	29.4
	Dust/fumes	28	20.6
	URTI/cold weather/dust	60	44.1
	Cigarette	0	0
	Unknown triggers	8	5.9

### Level of asthma control of the study participants

Based on the results of the 5-point Likert-type rating scale asthma control test, the scores ranged from 5 to 25. In this study 94 (69.1%) scored 19 or above, indicating controlled asthma, while the remaining 42 (30.9%) were found to have uncontrolled asthma. Among those with controlled asthma, 50 (53%) were male and 44 (47%) were female. Additionally, 48 (51%) of the children with controlled asthma were aged 7–10 years, 38 (40%) were aged 10–14 years, and only 8 (9%) were aged 14–17 years (Table 3).

**Table 3:** Asthma control status of asthmatic children at selected public hospitals in Addis Ababa, Ethiopia, 2024 (*N* = 136).

		Control asthma	Uncontrolled asthma
Gender	Male	50 (53.2%)	22 (46.8%)
	Female	22 (52.3%)	20 (47.6%)
Age	7–10	48 (51.1%)	36 (85.7%)
	10–14	38 (40.4%)	2 (4.7%)
	14–17	8 (8.5%)	4 (9.5%)

### Quality of life of study participants

According to the results obtained from the pediatric quality of life assessment tool, this study revealed that a significant proportion of children 51% (95% CI: 43–60%), experienced minimal to no impairment in their activity-related quality of life. Furthermore, 74 [54.45% (95% CI: 46–63%)] were also found to have minimal to no impairment in symptoms-related quality of life. Similarly, approximately 53% (95% CI: 44–61%) of these children exhibited minimal to no impairment in emotion-related quality of life. The overall good quality of life of the study participants was found to be 73 [54% (95% CI: 46–63%)] (Table 4).

**Table 4:** Asthma-related quality life of children at selected public hospitals in Addis Ababa, Ethiopia, 2024 (*N* = 136).

	AQOAL	SQOAL	EQOAL
Minimal impairment	70 (51.5%)	74 (54.4%)	72 (52.9%)
Moderate impairment	66 (48.5%)	62 (45.6%)	62 (47.1%)

AQOAL, Activity-related quality of life; SQOAL, Symptom-related quality of life; EQOAL, Emotion-related quality of life.

### The distribution of the degree of impairment reported by the patients in each PAQOL by levels of asthma control

Among children with a good quality of life, 106 (78%) had controlled asthma, whereas 84 (62%) of those with moderate impairment in activity-related quality of life had uncontrolled asthma. In the group with controlled asthma, the proportions of minimal and moderate impairment in symptom, activity, and emotion-related quality of life were equal, at 58% for minimal impairment and 43% for moderate impairment. Conversely, among children with uncontrolled asthma, the highest percentage of moderate impairment was observed in activity-related quality of life (62%), followed by emotion-related quality of life (57%) and symptom-related quality of life (52%).

### Factors affecting the quality of life

In this study, a total of 12 variables were analyzed. In the bivariable analysis, factors such as gender, age of the child, mother’s education level, family history of asthma, duration of asthma, severity of asthma, body mass index, comorbidity, and asthma control were identified as candidates for multivariable analysis. However, in the multivariable analysis, the educational status of the mother/caregiver, family history, duration, severity of asthma, asthma control, and comorbidity were found to be statistically significant at *p* < 0.05.

The study revealed that children with no formal education of caregivers were 1.39 times [AOR: 1.39 (1.80–10.69)] more likely to have a poor quality of life than those with above secondary education of caregivers. Furthermore, the odds of having poor quality of life increased about three times in the children with a family history of asthma [AOR: 2.51 (1.46–4.29)].

Children with a duration of asthma (SD) ≥25 (14) months nearly threefold increased their odds of poor quality of life than their counterparts [AOR: 3.47 (1.89–6.39)], and children whose asthma was uncontrolled 2-fold increased their odds of poor quality of life than those children with controlled asthma [AOR: 2.31 (1.17–4.56)].

Moreover, children with moderate persistent asthma had about three times higher odds [AOR: 3.40 (1.86–6.30)] poor quality of life, than those with mild persistent, while those with comorbidity nearly two times higher odds with poor quality of life [AOR: 2.40 (1.40–4.20)] compared to those who had not (Table 5).

**Table 5:** Factors associated with quality of life of asthmatic children at selected public hospitals in Addis Ababa, Ethiopia, 2024 (*N* = 136).

Variable	Categories	Quality of life		COR (95% CI)	AOR = (95% CI)	*p*-value
		Poor (%)	Good (%)			
Gender	Female	30 (46.9%)	34 (53.1%)	1	1	
	Male	32 (44.4%)	40 (55.6%)	0.91 (0.76–5.45)	1.31 (0.17–4.56)	0.116
Age of the child	7–10	36 (42.9%)	48 (57.1%)	1.03 (0.75–1.80)	0.85 (0.62–1.50)	0.42
	10–14	20 (50%)	20 (50%)	0.64 (0.57–0.95)	0.58 (0.47–1.02)	0.38
	14–17	6 (50%)	6 (50%)	1	1	
Educational status of the caregiver	No formal education	32 (54.8%)	26 (47.2%)	5.3 (2.04–11.50)	1.39 (1.80–10.70)**	0.001
	Primary	22 (52%)	20 (48%)	1.44 (0.60–3.90)	0.82 (0.35–2.15)	0.49
	Secondary	5 (23.6%)	15 (75.4%)	1.52 (0.95–2.45)	1.19 (0.67–2.12)	0.559
	Above secondary	3 (20%)	13 (80%)	1	1	
Family history of asthma	Yes	27 (78.9%)	7 (21.1%)	5.36 (3.47–8.30)	2.51 (1.46–4.30)**	0.001
	No	43 (42%)	59 (58%)	1	1	
Duration of asthma	< 25 ± 14 months	15 (25%)	45 (75%)	1	1	
	≥25 ± 14 months	43 (57%)	33 (43%)	3.9 (2.4–8.1)	3.47 (1.89–6.40)**	<0.001
Bodi mass index	Underweight	32 (51.6%)	30 (48.4%)	1	1	
	Normal weight	20 (33.3%)	40 (66.7%)	0.43 (0.29–2.73)	1.24 (0.52–2.98)	0.626
	Overweight	10 (71.4%)	4 (28.6%)	2.1 (0.7–3.02)	0.755 (0.21–2.78)	0.672
Asthma control	Controlled	5 (9.5%)	22 (24.7%)	1	1	
	Uncontrolled	43 (90.5%)	66 (75.3%)	2.9 (1.76–5.4)	2.31 (1.17–4.56)*	0.016
Severity of asthma	Mild persistent	18 (22.8%)	63 (77.2%)	1	1	
	Moderate persistent	41 (75.9%)	13 (24.1%)	11 (6.76–16.7)	3.4 (1.86–6.30)**	<0.001
Comorbidity	Yes	82 (62%)	51 (38%)	3.21 (1.8–6.3)	2.4 (1.40–4.20)**	0.001
	No	1 (33%)	2 (67%)	1	1	

*p < 0.05, **p < 0.01. AOR, Adjusted Odds Ratio; COR, Crude Odds Ratio; CI, Confidence Interval.

## Discussion

In this study, 46% [95% CI: 37.6–54.4%] of asthmatic children were found to have a poor quality of life. Factors such as the educational status of the mother/caregiver, family history, duration, severity of asthma, asthma control status, and comorbidity status significantly affected their quality of life.

The prevalence of poor quality of life in this study is consistent with similar studies conducted in the Amhara region 40.8% (19), and 49% in Palestine (16). However, it was lower compared to 61% in Nigeria (20), 59% in Egypt (18), and 60% in Peru (21). This variation could be attributed to differences in the study participants, variation in the study period, study setting, and most of the study participants in this study were from urban areas and had access to health services and their asthmatic status can be controlled and the controlled asthma leads the higher quality of life.

Regarding factors associated with the quality of life, children whose caregivers lack formal education were more likely to have a poor quality of life compared to those with above secondary education. This finding was found to be consistent with previous studies conducted in Peru (21), Nigeria (20), and Egypt (18). The possible reason behind this could be that Caregivers who lack formal education may rely more on traditional beliefs that discourage modern medicine or encourage ineffective remedies. Limited education can also hinder their access to reliable asthma management information due to socioeconomic barriers, including restricted internet access and fewer community resources. Additionally, lower educational attainment often limits employment opportunities, leading to lower household income, which can restrict access to nutritious food, safe housing, and healthcare all essential for effective asthma management.

Furthermore, having a family history of asthma increases about three times the odds of poor quality of life. These findings were supported by studies conducted in Ethiopia (22), and Egypt (18). The possible justification might be due to both genetic and environmental factors.

Children with an average duration of asthma (SD) ≥25 (14) months nearly threefold increased their odds of poor quality of life than their counterparts, which was found to be concurrent with studies conducted in Palastin (16), Egypt (18), and Nigeria (20). A possible explanation might be its multifaceted impact on physical health, emotional well-being, social interactions, treatment burden, and potential long-term complications.

Additionally, Children with uncontrolled asthma were twice as likely to have a poor quality of life compared to those with controlled asthma. This aligns with studies conducted in the Middle East and North Africa (23), Palestine (16), and Nigeria (20). The reason behind this could be children with uncontrolled asthma may face disruptions in daily activities, frequent exacerbations requiring hospitalization, and psychological challenges such as anxiety and depression.

Moreover, children with moderate persistent asthma had about three times higher odds of poor quality of life, than those with mild persistent. These findings were found to be consistent with previous studies conducted on Indonesia (24), and Palestine (16). The possible, justification found to be children with moderate persistent asthma could have limitations in daily activities, frequent symptoms and exacerbations, medication side effects, emotional impact, and social challenges, which increased their overall poor quality of life.

Children with comorbidity with their asthma had nearly two times higher odds of having a poor quality of life compared to those who had not. These findings were supported by studies conducted in Palestine (16), and Addis Ababa (22). The possible reason behind this could be the presence of one or more additional health conditions that can exacerbate the negative effects of asthma on quality of life, and managing multiple health conditions simultaneously can be challenging for both children and their caregivers.

The study’s results have important clinical implications, for healthcare professionals to identify patients at risk and implement targeted interventions to improve their overall well-being. By addressing these modifiable factors, clinicians may be able to help asthma patients better manage their symptoms and enhance their day-to-day functioning.

## Strengths and limitations of the study

### Strengths

The study’s strength lies in its inclusion of factors such as severity and duration of asthma to assess their potential impact on the quality of life of the study participants which was not the focus of previous studies in Ethiopia.

### Limitations

The cross-sectional nature of this study makes causal relationships between dependent and independent variables impossible. Since the study was based on self-reports, the respondents might be prone to social desirability bias.

## Conclusion

This study revealed that almost half of the respondents were found to have a poor quality of life. Factors such as having no formal education of caregivers, having a family history, duration and severity of asthma, having uncontrolled asthma, and having comorbidity were significantly associated with poor quality of life.

Based on the findings of this study, it is recommended to implement education and awareness programs specifically designed for caregivers who have no formal education. Furthermore, healthcare professionals should conduct comprehensive assessments of family medical histories to ensure appropriate care. The Addis Ababa health office should emphasize the importance of implementing effective strategies for managing asthma, with a particular focus on symptom control, screening, and managing comorbidities, and promoting community-based support programs. Lastly, it is suggested that further research be conducted to explore other potential determinants and to focus on comparative and prospective studies.

## Data Availability

The raw data supporting the conclusions of this article will be made available by the authors, without undue reservation.
